# First Draft Genome Sequence of a *Polymyxa* Genus Member, *Polymyxa betae,* the Protist Vector of Rhizomania

**DOI:** 10.1128/MRA.01509-18

**Published:** 2019-01-10

**Authors:** Alain Decroës, Magnalena Calusinska, Philippe Delfosse, Claude Bragard, Anne Legrève

**Affiliations:** a Earth and Life Institute, Applied Microbiology, Phytopathology, Université Catholique de Louvain, Louvain-la-Neuve, Belgium; b Environmental Research and Innovation Department, Luxembourg Institute of Science and Technology, Belvaux, Luxembourg; Vanderbilt University

## Abstract

Polymyxa betae belongs to the *Plasmodiophorida* (Phytomyxea, Rhizaria). Here, we report the first draft genome sequence of a member of the Polymyxa genus, which includes two obligate root endoparasite species, vectors of important soilborne plant viruses. The genome assembly was represented by 1,001 contigs, with a cumulated length of 27,085,946 bp.

## Abstract (precis)

Polymyxa betae belongs to the *Plasmodiophorida* (Phytomyxea, Rhizaria). Here, we report the first draft genome sequence of a member of the Polymyxa genus, which includes two obligate root endoparasite species, vectors of important soilborne plant viruses.

## ANNOUNCEMENT

Polymyxa betae and Polymyxa graminis belong to the *Plasmodiophorida,* a protist order which includes obligate root endoparasites of plants (1, 2). Although Polymyxa sp. infections are symptomless, these species are considered a real threat because of their vectorial capacity for viruses (3–5). Molecular studies of these organisms are difficult due to their biotrophic nature. Recently, they have been boosted with the publication of Plasmodiophora brassicae and Spongospora subterranea genomes and transcriptomes (6–9). Here, we present the first full draft genome sequence of P. betae, the vector of *Beet necrotic yellow vein virus*, the agent of rhizomania, *Beet soilborne mosaic virus, Beet soilborne virus*, and *Beet virus Q*.

Three-week-old Beta vulgaris of the fully sequenced monogerm DH line KWS2320 (10) were inoculated with zoospores of a P. betae single sporosorus (aggregation of resting spores) strain, and grown for 14 weeks in an automated immersion system (11). Heavily infested roots were harvested, surface disinfected with 1 N NaOH and 0.1% SDS, and the sporosori were extracted in a Virtis blender. After sequential filtration steps (500, 200, and 100 µm), sporosori were disinfected with 70% ethyl alcohol (EtOH) and 0.5% antiseptic liquid (Dettol), rinsed three times in sterile distilled water, resuspended in 100 µg/ml cycloheximide (Carl-Roth), 50 µg/ml tetracycline (Sigma-Aldrich), and 100 µg/ml gentamicin sulfate (Sigma-Aldrich) and kept at 4°C. Sporosori were then purified by two 3-h-long centrifugations at 4,500 × *g* on a 0 to 30% continuous gradient of HS-40 colloidal silica (Ludox; Sigma-Aldrich), treated with 50 U/ml RQ1 DNase (Promega) at 37°C for 1 h, and rinsed five times. The resulting material was ground in liquid nitrogen with mortar and pestle, and DNA was extracted following a cetyltrimethylammonium bromide (CTAB) extraction procedure (12).

One library was prepared with the TruSeq Nano DNA sample preparation kit and paired-end sequenced using an Illumina MiSeq instrument and the reagent kit version 3, with a sequencing length of 2 × 250 bp, yielding 12,292 Mb. After adapter trimming with Trimmomatic version 0.32 (13), 49,092,018 reads were quality trimmed and assembled using CLC Genomics Workbench version 9.5.2, resulting in 12,450 contigs (≥1 kbp). Contigs were binned using MetaBAT version 0.32.4 (14), resulting in 47 bins, which were assessed for genome completeness and contamination and assigned bin higher-level taxonomy with CheckM version 1.0.11 (15). Bacterial metagenome-assembled genomes (MAGs) and putative viral (phage) genomes were discarded following further BLAST annotation against the NCBI nr database. Raw reads originating from B. vulgaris KWS2320 leaf DNA (SRA accession numbers SRR869626 and SRR869627) were mapped to the resulting contigs with CLC Genomics Workbench version 9.5.2 to further remove plant DNA contamination. The remaining contigs were visualized with ICoVeR (16), and based on the GC content, coding density, contig coverage, and the tetranucleotide composition, a subset of 1,001 contigs was selected. This final potentially free-of-contamination assembly had a total length of 27,085,946 bp, a coverage of 225×, and an *N*_50_ value of 61,339 bp (*L*_50_, 106). The GC content is 45.1%, a percentage similar to that of S. subterranea (45.7%) but lower than that of P. brassicae (58.5%) (6, 8). Genome completeness was assessed using CEGMA version 2.5 (17). Of the 248 CEGMA eukaryotic core genes, 91.3% were completely (89.1%) or partially (2%) recovered in the assembly. The k-mer genome size estimation using the 17-mer coverage distribution inferred with Jellyfish version 2.0 (18) led to a complete genome size of 29,353,583 bp.

### Data availability.

This whole-genome shotgun project has been deposited in DDBJ/ENA/GenBank under the accession number RBZT00000000 and BioProject number PRJNA494670. Raw reads are available under SRA accession number SRR8105394. The version described in this paper is the first version, RBZT01000000.
